# Identification of Plasma Metabolites Responding to Oxycodone Exposure in Rats

**DOI:** 10.3390/metabo15020095

**Published:** 2025-02-04

**Authors:** Thao Vu, Suneeta Godbole, Lieselot L. G. Carrette, Lisa Maturin, Olivier George, Laura M. Saba, Katerina Kechris

**Affiliations:** 1 Department of Biostatistics & Informatics, Colorado School of Public Health, University of Colorado Anschutz Medical Campus, Aurora, CO 80045, USA; 2 Department of Psychiatry, School of Medicine, University of California San Diego, La Jolla, CA 92093, USA; 3 Department of Pharmaceutical Sciences, Skaggs School of Pharmacy and Pharmaceutical Sciences, University of Colorado Anschutz Medical Campus, Aurora, CO 80045, USA

**Keywords:** plasma metabolomics, prescription opioids, oxycodone exposure, rodent model

## Abstract

Background: Oxycodone has an elevated abuse liability profile compared to other prescription opioid medications. However, many human and rodent metabolomics studies have not been specifically focused on oxycodone. Objectives: Investigating metabolomics changes associated with oxycodone exposure can provide insights into biochemical mechanisms of the addiction cycle and prognosis prediction. Methods: Plasma samples from 16 rats at pre-exposure and intoxication time points were profiled on the Metabolon platform. A total of 941 metabolites were characterized. We employed a k-Nearest Neighbor imputation to impute metabolites with low levels of missingness and binarized metabolites with moderate levels of missingness, respectively. Results: Of the 136 binarized metabolites, 6 showed differential abundance (FDR < 0.05), including 5 that were present at pre-exposure but absent at intoxication (e.g., *adenine*), while *linoleamide (18:2n6)* exhibited the opposite behavior. Among the 798 metabolites with low levels of missingness, 364 showed significant changes between pre-exposure and intoxication (FDR < 0.01), including *succinate*, *oleamide*, and *sarcosine*. We identified four pathways, including *tryptophan metabolism,* that were nominally enriched among the metabolites that change with oxycodone exposure (*p* < 0.05). Furthermore, we identified several metabolites that showed nominal correlations with the Addiction Index (composite of oxycodone behaviors): 17 at pre-exposure and 8 at intoxication. In addition, the changes in abundance between pre-exposure and intoxication time points of 9 metabolites were nominally correlated with the Addiction Index, including *sphingomyelins*, *methylhistidines*, and *glycerols*. Conclusions: In summary, not only were we able to capture oxy-induced changes in metabolic pathways using easily accessible blood samples, but we also demonstrated the potential of blood metabolomics to better understand addiction liability.

## 1. Introduction

In the United States, the misuse and dependence on prescription opioids have escalated into a public health crisis. Approximately 10 million individuals were reported to misuse prescription opioids, and the toll from opioid overdose reached nearly 106,699 lives in 2021 [1]. Florence et al. estimated the overall economic burden of opioid use disorder and fatal opioid overdose in the US for 2017 to be $1.02 trillion, with major components attributed to the reduced quality of life and the value of life lost [2].

The Dole–Nyswander theory during the 1960s suggested the initial step of addiction was attributed to persistent metabolic disruptions following the first instance of narcotic drug use [3]. By studying small molecules, known as metabolites, that are end-products of enzymatic reactions, metabolomics aims to provide a more direct assessment of the underlying biological processes that respond to external stimuli, environmental stress, or genetic adaptions [4]. In essence, metabolomics not only enables the identification of endogenous disease biomarkers but also facilitates the detection of exogenous metabolites. Through the study of perturbations in metabolic status associated with addiction profiles and drug exposure, clinically relevant biomarkers can be identified, thereby deepening our understanding of the underlying etiology of drug addiction [3]. For instance, by comparing metabolomics profiles across different addiction phases, including current drug abusers, withdrawal periods, durations of abstinence, relapse, or no drug use, biomarkers associated with the addiction trajectories can be identified [3,5]. An example of the effect of drug exposure includes a metabolomics study on brain tissues from morphine- versus saline-treated monkeys to identify metabolites such as *taurine, lactic acid*, and *methionine*, with perturbed concentrations in the morphine-treated monkeys relative to the controls [6].

Oxycodone is a potent analgesic from the opioid family, widely used for the treatment of moderate to severe pain. Chemically resembling morphine, oxycodone was initially synthesized in Germany in 1916 and has been increasingly prescribed for alleviating chronic non-cancer pain such as diabetic neuropathy [7,8], back pain, or osteoarthritis [9,10] due to its better analgesic efficacy with fewer deleterious physical side effects. Notably, oxycodone has been shown to have a significantly elevated abuse liability profile compared to other opioids, primarily due to high likability scores and fewer negative subjective ratings [11,12,13]. Oxycodone, ranked as the third leading opioid after heroin and fentanyl, has contributed to 33,154 overdose deaths in the US between 2011 and 2016, averaging between 4967 and 6199 annually. In 2021, the number of overdose deaths increased to 13,618 [14]. In terms of morbidity, an estimated 49,609 emergency cases per year were linked to non-medical use of oxycodone products, according to the National Electronic Injury Surveillance System-Cooperative Adverse Drug Event Surveillance (NEISS-CADES) data [13].

Despite the significant public health concerns surrounding oxycodone, many human and rodent metabolomics studies have not specifically targeted oxycodone [15,16,17,18]. For example, Li et al. [15] evaluated the urine metabolic profiles of 218 opium users and 80 non-users in the Golestan Cohort Study (GCS) to identify a set of endogenous compounds differentiating the two group users. In a different study, Zheng et al. [16] explored the impact of methamphetamine use on the serum and urine metabolic pathways in male Sprague Dawley rats to better understand its mechanisms for toxicity. To bridge the gap, we conducted a study to leverage plasma samples from the Oxycodone Biobank [19], a large repository of tissues and biofluid samples from heterogeneous stock (HS) rats designed to mimic the genetic diversity in the human population. Based on the plasma metabolomics data, we investigated changes associated with oxycodone exposure to provide insights into the biochemical mechanisms of the addiction cycle and the metabolic consequences of exposure related to this opioid.

## 2. Materials and Methods

Oxycodone Biobank: The Oxycodone Biobank [19] is a large repository containing more than 20,000 tissue and biofluid samples from approximately 1000 heterogeneous stock (HS) rats, which are highly recombinant animals, created by crossbreeding eight genetically diverse founder inbred strains to mimic the diversity observed within the human population. In particular, blood samples are collected longitudinally: before oxycodone exposure, during intoxication, during acute withdrawal, and after protracted abstinence. Each rat is fully characterized for addiction-like behaviors based on the well-validated models of intravenous oxycodone self-administration and the comprehensive characterization of addiction-related behaviors [20,21,22,23,24]. The paradigm has been shown to include phenotypes similar to 6 of the 11 criteria for substance use disorders (SUD) outlined in the DSM-5 [25], including most of the criteria required to diagnose severe use disorder in humans: (1) tolerance, (2) withdrawal, (3) substance taken in larger amount than intended, (4) unsuccessful efforts to quit, (5) considerable time spent to obtain the drug, and (6) continued use despite adverse consequences [19].

Oxycodone Biobank Drug Exposure: Oxycodone (Sigma-Aldrich) was dissolved in 0.9% sterile saline (Hospira) and administered at 150 mg/kg per infusion intravenously. This dose of oxycodone per infusion was selected based on previous studies and because it produces significant plasma oxycodone concentrations (40 ng/mL) [26]. Rats were familiarized and trained to self-administer during short access (ShA) and long access. There were 4 ShA sessions of 2 h and 14 LgA sessions of 12 h.

Plasma Samples: For this study, the Oxycodone Biobank [19] provided 32 plasma samples from 16 rats (8 females and 8 males; 2 samples per rat) at pre-exposure and intoxication time points. While the rats were anesthetized for the intravenous catheter surgery, 200- to 400-μL blood (pre-exposure) was collected through retroorbital bleed in EDTA-coated tubes, which were immediately inverted five times. Blood samples were processed within 1 h of collection by centrifugation at 2000× *g* at room temperature (RT) for 10 min to pellet the erythrocytes. The supernatant plasma was immediately transferred into a fresh tube, scored for quality on a scale from 0 to 5, snap-frozen on dry ice, and stored at –80 °C. Samples were shipped on dry ice overnight to the Metabolon facility in North Carolina for metabolite characterization. Of the 32 plasma samples, one sample (M1454 at baseline) was excluded as it did not pass Metabolon QC standards.

Metabolomics Data Generation: We used the well-established Metabolon Global Discovery platform to generate untargeted metabolomic profiles. The platform is based on ultra-performance (UP) liquid chromatography (LC)-mass spectrometry (MS), which is an inherently semi-quantitative, highly sensitive technology that measures the relative quantity of an individual metabolite as expressed by the metabolite’s peak intensity variations in comparative samples. Samples received from UCSD were inventoried and immediately stored at −80 °C until processed. Samples were divided into five fractions: 2 for analysis by two separate reverse phases (RP)/UPLC-MS/MS methods with positive ion mode electrospray ionization (ESI), 1 for analysis by RP/UPLC-MS/MS with negative ion mode ESI, 1 for analysis by hydrophilic interaction chromatography (HILIC)/UPLC-MS/MS with negative ion mode ESI, and 1 sample reserved for backup. Several types of controls were analyzed in concert with the experimental samples that allowed instrument performance monitoring and aided chromatographic alignment. A total of 941 annotated metabolites were characterized by Metabolon.

**Figure 1:**
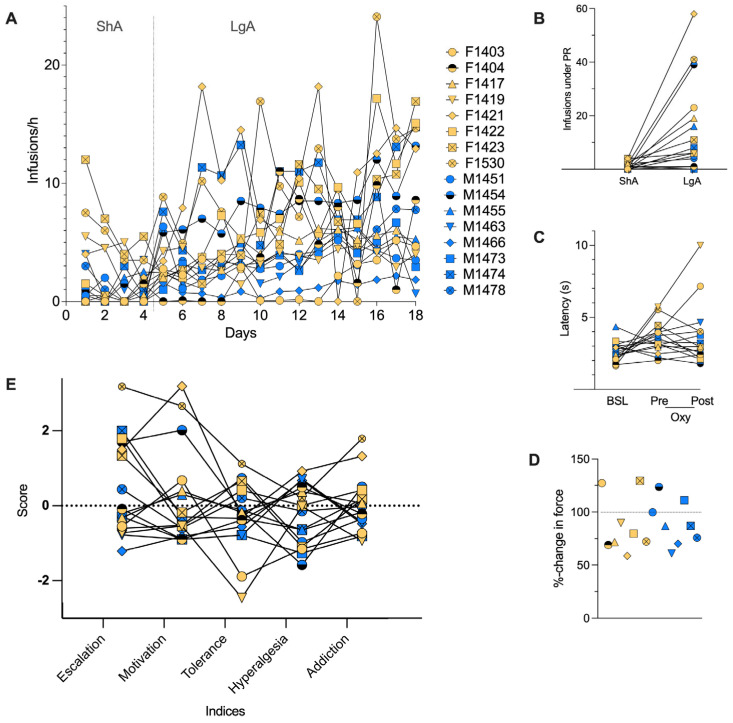
Individual differences in behavioral measures across 16 rats including 8 females (yellow) and 8 males (blue). (**A**) Escalation of oxycodone intake through self-administration of individual rats under a fixed ratio after short access (ShA) and long access (LgA). There are 4 ShA sessions of 2 h and 14 LgA sessions of 12 h. (**B**) Motivation through self-administration under a progressive ratio after ShA and LgA. (**C**) Analgesia and tolerance through tail immersion test evaluating the latency to lift the tail out of hot water at baseline (BSL) before ShA session and before LgA session. (**D**) Hyperalgesia through Von Frey test measuring force for paw lifting at baseline and in withdrawal 12 h after the last LgA session. (**E**) Indices for four phenotypes: escalation, motivation, tolerance, and hyperalgesia. The addiction index is calculated by averaging the four behavioral indexes.

Addiction Index: To evaluate addiction-like behaviors in individual rats, we used the Addiction Index [19,26], which was calculated as an average z-score that is a composite (Figure 1E) of four phenotypic indices including escalation of oxycodone intake, motivation for oxycodone, tolerance to the analgesic effects of oxycodone, and withdrawal-induced hyperalgesia (Figure 1A–D). Behavioral z-scores and indices were calculated to control for sex and cohort effects and the scale of behavioral paradigm outputs (see Carrette et al. [19] for details). High Addiction Index values correspond to severe oxycodone addiction-like behaviors, while low values indicate resilience to such behaviors.

Metabolomics Data Pre-Processing: Normalization was performed for each metabolite by dividing the intensity value of each metabolite by its median intensity across samples. Among the 941 annotated metabolites, there were 798 metabolites that were missing abundance levels in less than 20% of samples, referred to as low-missing metabolites. A smaller set of 136 metabolites had 20–80% of samples with missing values, referred to as moderate-missing metabolites. Finally, there were 7 metabolites missing >80% of samples. After normalization, we imputed the missing values based on the degree of missingness, as follows:

- We removed 7 metabolites with missing values in more than 80% of samples.
- For moderate-missing metabolites, we binarized the values into presence/absence (1/0) categories, i.e., whether the level of the metabolite was quantifiable or not.
- For low-missing metabolites: we used k-Nearest Neighbor (k = 10) to impute missing values using the R package ‘impute’ [27], followed by log-transformation. We utilized Principal Component Analysis (PCA) and relative log expression plots to explore the data and identify potential outliers or mislabeled samples.

After examining the PC score plot, we identified that the time point labels (pre-exposure vs. intoxication) for one rat (F1530) were switched (Figure S1). We corrected this before proceeding to subsequent analyses. For each sample, a median signal across all metabolites was calculated. Using these median signals, we computed a z-score for each sample. One sample (M1466 at intoxication) had a z-score greater than 3.5 standard deviations from the median of all samples; it was removed. Hierarchical clustering was used to explore similarities in the metabolomic profiles of individual rats across the two time points. Pairwise correlation coefficients between samples were used as distances. The distance matrix was then hierarchically clustered using complete-linkage clustering.

Analysis Methods: Using the R software version 4.4.0, differential abundance across time points (pre-exposure and intoxication) was conducted using a paired t-test for the low-missing metabolites and McNemar’s Chi-squared test for the moderate-missing metabolites to account for the paired data structure. Pearson correlation analyses examined the association between metabolites at each time point and the difference in metabolite abundances between time points with the Addiction Index. Note that since we removed one sample (M1454 at baseline) due to low QC and another outlier (M1466 at intoxication), we have *n* = 14 for any paired analyses and *n* = 15 for time point-specific analyses.

Pathway Analysis: Metabolon provides annotation of 941 metabolites at 9 super-pathways and 110 sub-pathways. Overrepresentation of Metabolon super- and sub-pathways was conducted using Fisher’s Exact test. *p*-values were adjusted for multiple comparisons using a false discovery rate (FDR).

## 3. Results

### 3.1. Metabolite Differences Between Pre-Exposure and Intoxication

**Figure 2:**
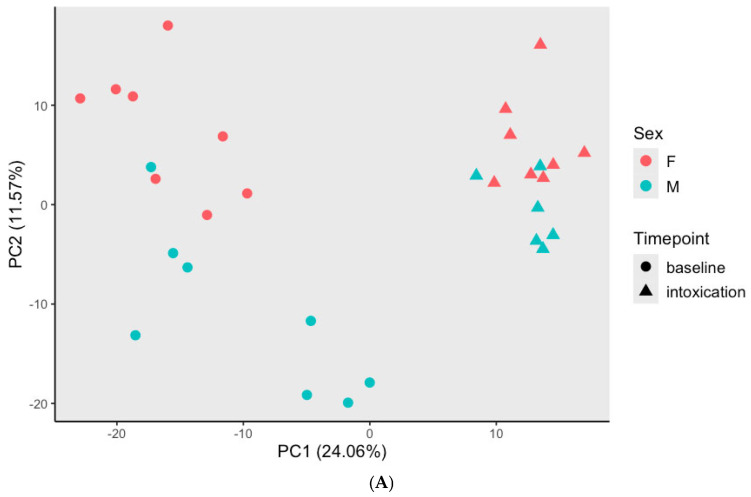

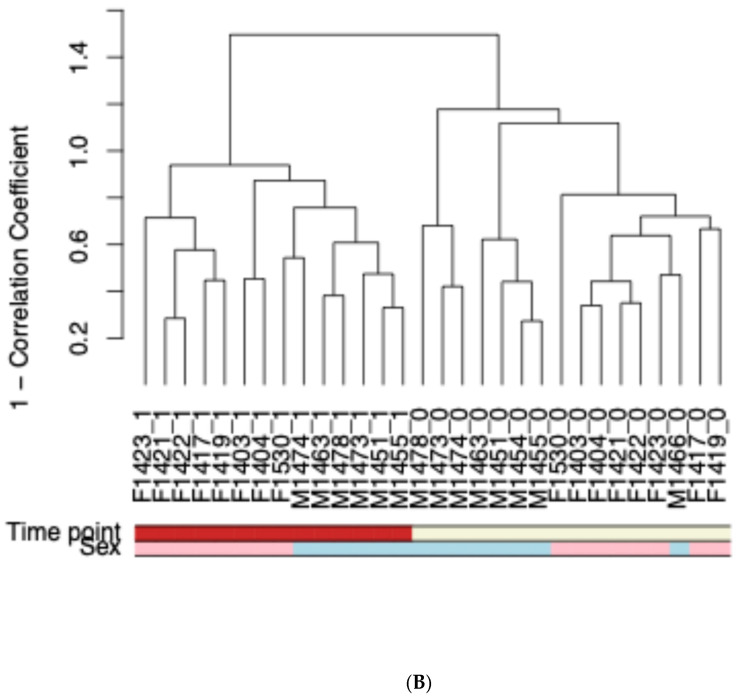
(**A**) PCA scores plot separate by sex and time point. PC1 separates pre-exposure and intoxication samples (*p* < 0.001). PC2 separates female and male samples (*p* < 0.001). (**B**) Hierarchical clustering using pairwise correlation distance between samples with complete linkage. Clusters are separated by time point: baseline (beige), intoxication (red), and by sex: female (pink), male (blue).

**Figure 3:**
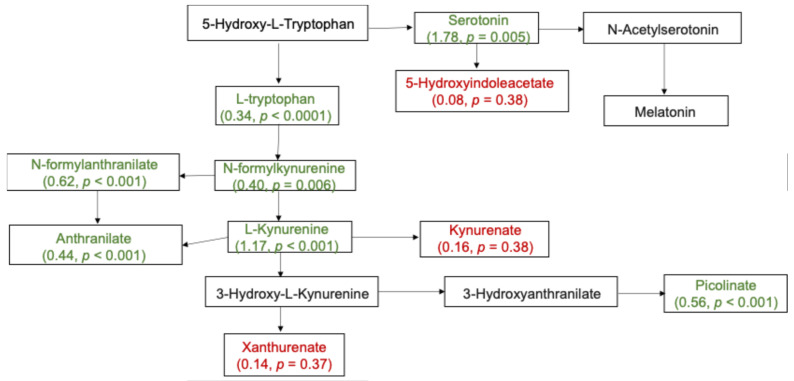
Tryptophan metabolism sub-pathway. Metabolites highlighted in green show a significant increase in abundance level at intoxication compared to pre-exposure. Metabolites in red did not have significant differential responses between the two time points. Metabolites in black were not measurable in our study.

The PCA scores plot indicates that the largest source of variation was time point, with the first principal component clearly separating pre-exposure samples and intoxication samples (Figure 2A, *p* < 0.001). The second largest source of variation (represented by PC2) was sex, demonstrated by the separated male and female clusters at each time point (Figure 2A, *p* < 0.001). This hierarchical relationship is further demonstrated in Figure 2B. Of the 136 binarized moderate-missing metabolites, we identified 6 metabolites (Table S1) with differential abundance (FDR < 0.05), including 5 being present at pre-exposure but absent at intoxication (i.e., *adenine*, *cefazolin, indoleacetoylcarnitine*, *4-hydroxybutyrate (GHB)*, *2,2′-methylenebis(6-tert-butyl-p-cresol))*, and *linoleamide (18:2n6)*, which was present at intoxication but absent at pre-exposure. Of the 798 low-missing metabolites, 364 changed between pre-exposure and intoxication time points (FDR < 0.01). *Oleamide, succinate,* and *sarcosine* (Table 1) ranked among the top differentially expressed metabolites based on the magnitude of change, in which *oleamide* demonstrated a significant increase in abundance at intoxication compared to pre-exposure, while *succinate* and *sarcosine* exhibited the opposite behavior. There were no statistically significant overrepresented pathways after adjustment for multiple comparisons. However, eight pathways (Table S2), including *tryptophan metabolism* (increased at intoxication), were nominally significant (*p* < 0.05). Out of the 22 *tryptophan metabolism* sub-pathway metabolites measurable in this study, we identified 17 with significant changes between pre-exposure and intoxication (Figure 3, Table S2). Notably, 15 of these metabolites, including *tryptophan*, *serotonin*, and *kynurenine*, showed a significant increase in the abundance level at intoxication compared to pre-exposure.

### 3.2. Metabolites and the Addiction Index

**Figure 4:**
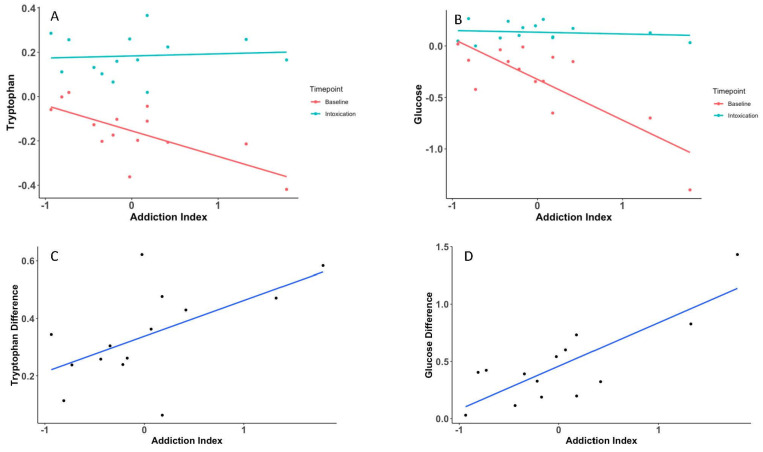
Plasma tryptophan (**A**) and glucose (**B**) levels at baseline and intoxication vs. the Addiction Index for individual rats. Changes in plasma tryptophan (**C**) and glucose (**D**) between intoxication and pre-exposure with the Addiction Index.

To evaluate metabolites associated with addiction-like behaviors, we used the Addiction Index [19,26], which was calculated as an average z-score of four phenotypic indices including escalation, motivation, tolerance, and hyperalgesia (see Figure 1 and Carrette et al. [19] for details). We identified 17 metabolites at pre-exposure and 8 at intoxication that were nominally correlated (*p* < 0.01) with the Addiction Index (Table S3). Notably, *tryptophan* was negatively correlated (−0.71, *p* = 0.005) with the Addiction Index at the pre-exposure time point, but not at intoxication (0.08, *p* = 0.7) (Figure 4A). Nine metabolites, including *sphingomyelins*, *methylhistidines,* and *glycerols*, exhibited changes in abundance levels across the time points that were nominally associated with the Addiction Index. *Glucose* was nominally associated with the Addiction Index both at pre-exposure (−0.80, *p* = 0.005) (Figure 4B) and using the change between pre-exposure and intoxication (0.81, *p* = 0.0004) (Figure 4D). Similarly, the change in plasma tryptophan between intoxication and pre-exposure was nominally associated with the Addiction Index (0.59, *p* = 0.028) (Figure 4C).

## 4. Discussion

Metabolomics plays an important role in identifying biomarkers associated with opioid addiction dependence, withdrawal, and relapse, thereby providing valuable insights into underlying biological processes. Although oxycodone has an elevated abuse liability profile compared to other opioids, many previous human and rodent metabolomics studies have not specifically focused on this substance [15,17,18]. In this study, we utilized a rat model to investigate changes in the metabolomics profiles of plasma samples in the Oxycodone Biobank to identify differential abundance metabolites across pre-exposure and intoxication time points. Additionally, by correlating metabolite abundances at the two time points with the Addiction Index, we further explored specific metabolic changes that might indicate a rat’s susceptibility or resilience to compulsive oxycodone use. This approach allowed us to identify metabolites related to an individual’s vulnerability to oxycodone addiction, providing a deeper understanding of the biological mechanisms underlying predisposition to this opioid.

We observed that *linoleamide* was present at intoxication but absent before the oxycodone exposure. In a human metabolomics study of plasma, *linoleamide* was increased in the plasma of chronic alcoholics compared to controls [28]. In our study, *adenine* was found to be present at pre-exposure but absent at intoxication. Similarly, in a study by Li et al. [15], *adenine* was shown to lower in urine samples from opium users relative to the controls. Furthermore, among the metabolites exhibiting significantly differential abundances between pre-exposure and intoxication, *oleamide, succinate,* and *sarcosine* ranked among the top with large estimated absolute differences in abundance levels across the two time points. In a study by Choi et al. [29], *oleamide* was found to indicate a modulation of central neurotransmission. Notably, the authors observed a reduction of *oleamide* in rat hair following methamphetamine self-administration, while our study revealed an increase in its abundance in plasma following oxycodone exposure. *Succinate* related to the Krebs cycle was shown to be lowered in opium users compared to the control group [15]. Similarly, *succinate,* related to energy metabolism, also exhibited a decrease in abundance level in methamphetamine-treated rats compared to the controls [30]. Likewise, our analysis indicated a decrease in *succinate* after oxycodone exposure. Additionally, urine levels of *sarcosine*, an intermediate in the metabolism of *choline* to *glycine*, were shown to be highly predictive of an opioid use disorder (OUD) positive diagnosis, which demonstrated an increase in chronic high opioid users diagnosed as OUD-positive compared to those without OUD [31]. Interestingly, in our study, we observed a decrease in plasma *sarcosine* levels after oxycodone exposure.

Even though we found no statistically significant overrepresented pathways after multiple comparison adjustments, we found pathways including *tryptophan metabolism* significant at the nominal level. The majority of the metabolites in the tryptophan metabolism sub-pathway, including *tryptophan*, a precursor to *serotonin*, *melatonin*, and *kynurenine,* were significantly elevated at intoxication. These *tryptophan* metabolites have been shown to have a variety of effects on the central nervous system and hold promise as potential treatment options for managing the compulsive abuse of addictive substances [32]. *Serotonin* deficits have been implicated in physical symptoms and emotional dysphoria following withdrawal from opioids [33]. *Melatonin* was observed to indicate the abnormalities in neurotransmission elicited by chronic heroin use [34]. Interestingly, recent findings have shown a decrease in *tryptophan* concentration in the plasma of morphine-addicted rats and mice [35,36], while an earlier study revealed a decrease in *tryptophan* abundance in rat blood plasma and an increase in rat brain after morphine administration [37]. However, a study by Kutchy et al. also showed distinct plasma metabolic signatures in morphine-tolerant mice compared to those induced by acute intake [35]. They found a significantly increased *tryptophan* level in the plasma of morphine-tolerant mice, which is consistent with our findings in the plasma of rats after oxycodone exposure. It is worthwhile for us to conduct further studies focusing on rat brain metabolomics to investigate the association between oxycodone addiction and disrupted brain energy metabolism.

In the correlation analysis with the Addiction Index, we found no metabolites with a significant correlation after adjusting for multiple comparisons. However, both *tryptophan* and *glucose* showed a nominal negative correlation with the Addiction Index during pre-exposure but not at intoxication. It is worth noting that opioids can impact glucose metabolism, and exposure to opioids has been linked to increased blood glucose levels [38]. Solis et al. examined the effects of oxycodone on oxygen and glucose levels in the nucleus accumbens (NAc) of awake, freely moving rats [39]. Their findings revealed a transient decrease followed by an increase in oxygen and glucose levels in the NAc with increasing doses of oxycodone. It is possible that brain glucose levels could rise when blood glucose levels increase [40]. Therefore, the observed change of direction in behavior-associated glucose levels in our plasma metabolomics study may induce the biphasic fluctuations of glucose levels in the brain.

## 5. Limitations and Future Work

Due to a limited sample size, our study did not investigate sex-specific differences in the vulnerability to oxycodone addiction. However, the principal component analysis (Figure 2A) revealed a separation in metabolomics profiles between the sexes, as shown by PC2. This suggests that oxycodone-induced metabolomics changes may differ between females and males. We have performed an additional differential analysis by sex, at both baseline and intoxication, using the top 50 metabolites contributing the most to PC2. Among these metabolites (Table S4), 17 and 8 showed significant differences between females and males at baseline and intoxication, respectively. Before oxycodone exposure, females had significantly higher levels of *biotin, citrate*, and *allantoin* compared to males (FDR < 0.05), while the difference was not significant at intoxication. Conversely, *carnitine* levels were significantly lower in females at intoxication (FDR < 0.05) but not at pre-exposure. *Pseudoridine* levels were significantly higher in females at both time points (FDR < 0.05); however, the magnitude of the difference was larger at baseline than at intoxication (6.47 vs. 4.07).

It is important to note that the top metabolites showing significant sex differences were not part of the *tryptophan metabolism* pathway and did not overlap with the oxycodone-induced metabolites identified in our study. As such, our conclusion remains unaffected. In future work with larger sample sizes, we will use an interaction model between sex and oxycodone exposure to investigate metabolic differences in response to oxycodone between females and males.

Furthermore, our study focused on plasma metabolites, which may not fully reflect the metabolic changes occurring in the brain. Further research on brain metabolomics is necessary to confirm how these plasma markers relate to neurological processes.

## 6. Conclusions

Our study leveraged unique samples from the Oxycodone Biobank to uncover plasma metabolic changes associated with oxycodone exposure. Using differential analysis, we identified many metabolites with significant changes in abundance between pre-exposure and intoxication time points, echoing previous substance abuse studies. Interestingly, our investigation of metabolites within the nominally significant *tryptophan metabolism* pathway showed some variations from patterns observed in studies of other opioids, such as morphine and methamphetamine. This raised the question of whether these observed changes in the plasma metabolites were unique to oxycodone exposure and could potentially serve as biomarkers for this high-liability opioid. In summary, this study highlights the significance of plasma metabolomics in understanding oxycodone-induced effects on metabolite levels and offers a valuable resource for future investigation into the relationship between blood metabolomics and brain biochemistry.

## Figures and Tables

**Table 1:** Top 5 metabolites with significant positive (top) and negative (bottom) changes in abundance levels between pre-exposure and intoxication.

Metabolite	Super Pathway	Sub Pathway	Estimate	Adjusted *p*-Value
Positive change: higher abundance at intoxication relative to pre-exposure				
oleamide	Lipid	Fatty Acid, Amide	4.74	<0.0001
3-phenylpropionate (hydrocinnamate)	Xenobiotics	Benzoate Metabolism	2.5	<0.0001
N-acetylkynurenine (2)	Amino Acid	Tryptophan Metabolism	2.47	<0.0001
cholate	Lipid	Primary Bile Acid Metabolism	2.45	0.0088
cinnamate	Xenobiotics	Food Component/Plant	2.01	0.0015
Negative change: lower abundance at intoxication relative to pre-exposure				
iminodiacetate (IDA)	Xenobiotics	Chemical	−2.63	<0.0001
succinate	Energy	TCA Cycle	−2.57	<0.0001
sarcosine	Amino Acid	Glycine, Serine and Threonine Metabolism	−2.16	<0.0001
sphinganine	Lipid	Sphingolipid Synthesis	−1.91	<0.0001
hexanoylcarnitine (C6)	Lipid	Fatty Acid Metabolism (Acyl Carnitine, Medium Chain)	−1.82	<0.0001

## Data Availability

Metabolomics data have been submitted to the NIH Common Fund’s National Metabolomics Data Repository (NMDR) website, the Metabolomics Workbench, https://www.metabolomicsworkbench.org (accessed on 14 January 2025, Study ID ST003673). Behavioral data are available upon request. Further inquiries can be directed to the corresponding author.

## Supplementary Materials

The following supporting information can be downloaded at: https://www.mdpi.com/article/10.3390/metabo15020095/s1. Table S1. Differential analysis results between pre-exposure and intoxication for binarized metabolites. Table S2. Differential analysis results between pre-exposure and intoxication for continuous metabolites. Table S3. Correlation analysis results of all metabolites with the Addiction Index at each time point. Table S4. Differential analysis results between females and males of top 50 metabolites contributing to PC2, at pre-exposure and intoxication. Figure S1. PCA scores plots before (left) and after (right) time point labels switching.
