# Supplementary figures and images for: Identification of Plasma Metabolites Responding to Oxycodone Exposure in Rats
Source: Metabolites. 2025 Feb 4;15(2):95. doi: 10.3390/metabo15020095 (PMC11857688; doi:10.3390/metabo15020095)

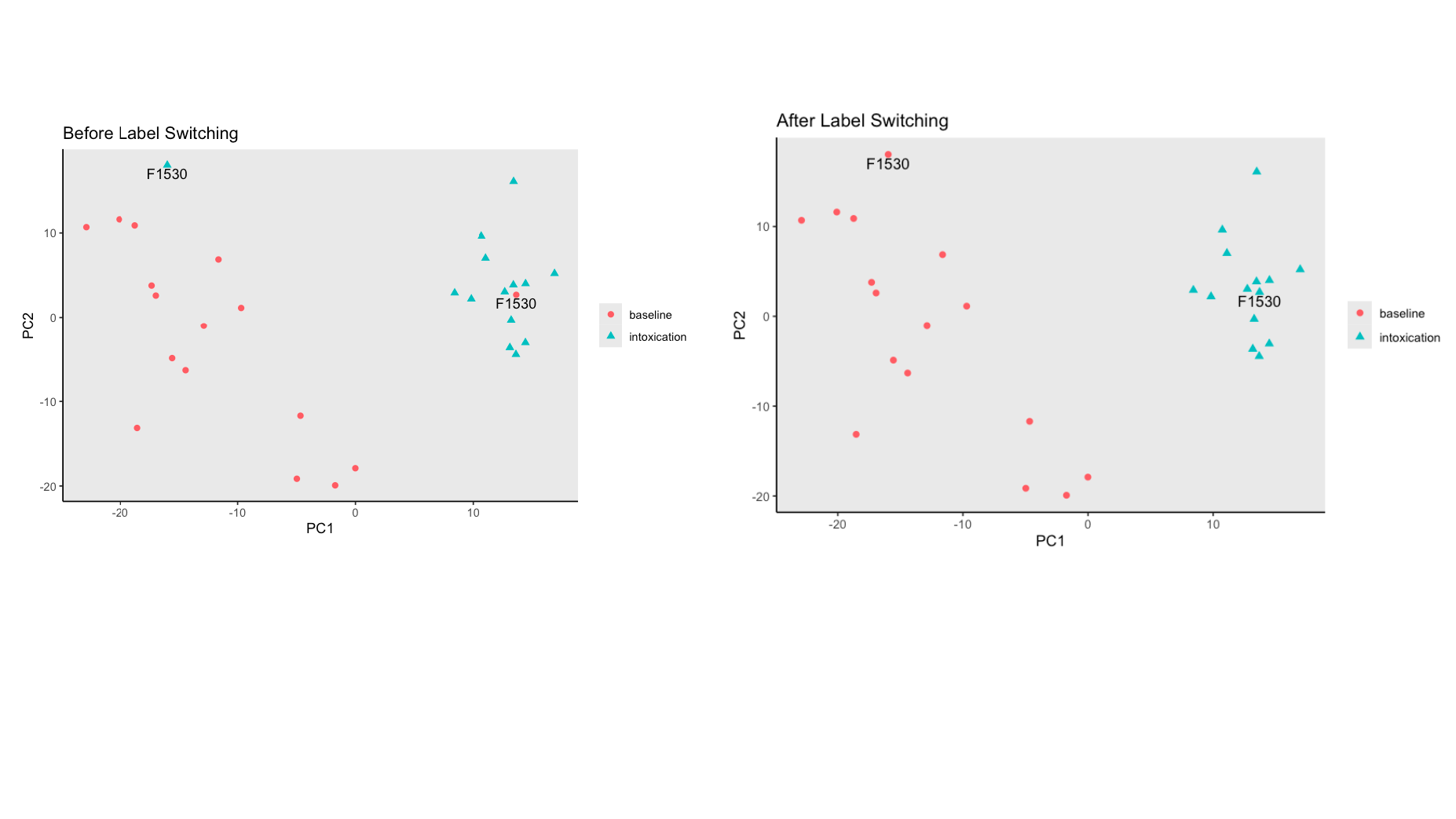

Supplement: Supplementary file 1 [file metabolites-15-00095-s001.zip › Figure_S1.png]
